# Integrative expression vectors with P*grac* promoters for inducer-free overproduction of recombinant proteins in *Bacillus subtilis*

**DOI:** 10.1016/j.btre.2020.e00540

**Published:** 2020-10-09

**Authors:** Dinh Thi Minh Tran, Trang Thi Phuong Phan, Thanh Thi Ngoc Doan, Thuoc Linh Tran, Wolfgang Schumann, Hoang Duc Nguyen

**Affiliations:** a Center for Bioscience and Biotechnology, University of Science, 227 Nguyen Van Cu Dist. 5, Ho Chi Minh City, Viet Nam; b Laboratory of Molecular Biotechnology, University of Science, 227 Nguyen Van Cu Dist. 5, Ho Chi Minh City, Viet Nam; c Department of Biology, Ho Chi Minh City University of Education, 280 An Duong Vuong, Dist. 5, Ho Chi Minh City, Viet Nam; d Agriculture and Food Technology Faculty, Tien Giang University, 119, Ap Bac, My Tho City, Tien Giang, Viet Nam; e Institute of Genetics, University of Bayreuth, D-95440, Bayreuth, Germany; f Department of Microbiology, University of Science, 227 Nguyen Van Cu Dist. 5, Ho Chi Minh City, Viet Nam; g Vietnam National University, Ho Chi Minh City, Viet Nam

**Keywords:** BgaB, β-galactosidase, LB, Luria broth, IPTG, isopropylthiogalactoside, MUG, methylumbelliferyl β-d-galactopyranoside, MCS, multiple cloning site, Inducer-free, Integrative expression vector, P*grac*01 promoter, P*grac*100 promoter, P*grac*212 promoter, pHT vector

## Abstract

Inducer-free integrative vectors are often used to create *B. subtilis* strains for industrial purposes, but employing strong promoters to produce high levels of recombinant proteins in *B. subtilis* results in high leaky expression that can hamper cloning in *Escherichia coli*. To overcome the problem, we used strong IPTG-inducible P*grac* promoters harboring *lac* operators to construct inducer-free integrative vectors able to integrate into the *B. subtilis* genome at either the *lacA* or the *amyE* locus, or both and examined their ability to repress the β-galactosidase (*bgaB*) gene in *E. coli* and to overexpress BgaB in *B. subtilis*. The P*grac*01 vectors could repress *bgaB* expression about 24-fold in *E. coli* to low background levels. The integrated P*grac*01-*bgaB* constructs exhibited inducer-free expression and produced 8% of total cellular proteins, only 1.25 or 1.75 times less compared with their cognates as plasmids. The stronger promoters, P*grac*100-*bgaB* and P*grac*212-*bgaB* yielded 20.9 % and 42 % of total intracellular proteins after 12 h of incubation, respectively. Incorporation of the P*grac*212-*bgaB* into both *amyE* and *lacA* loci resulted in BgaB expression up to 53.4 %. In conclusion, integrative vectors containing the P*grac* promoter family have great potential for inducer-free overproduction of recombinant proteins in *B. subtilis*.

## Highlights

• The new inducer-free integrative expression vectors could repress the reporter gene expression in the *E. coli* cloning strain, thereby facilitating the cloning step.
• The expression vectors carrying IPTG-inducible P*grac* promoters allow the production of the recombinant protein at high levels in *B. subtilis* in the absence of the inducer.
• The single-copy expression levels of integrative constructs, P*grac*01-*bgaB*, P*grac*100-*bgaB*, P*grac*212-*bgaB* could reach to % and 8%, 20.9 % and 42 % of total cellular proteins after 12 h incubation, respectively.
• The double integration of P*grac*212-*bgaB* into both *amyE* and *lacA* loci resulted in BgaB expression up to 53.4 %.

## 1 Introduction

*B. subtilis* is presently the best-characterized Gram-positive bacterium [1]. Its biochemistry, physiology and genetics have been studied intensively for more than fifty years [2]. As a result, a great deal of vital information concerning its transcription and translation mechanisms, genetic manipulation, and large-scale fermentation has been acquired [3]. In addition, it has a long history of industrial use because of its excellent growth on cheap carbon sources, and robustness under industrial conditions [4]. Another advantage is that *B. subtilis* is regarded as a Generally Recognized as Safe (GRAS) organism that lacks endotoxins and is non-pathogenic [5,6]. Furthermore, it has no significant bias in codon usage [3]. Therefore, it has become an ideal bacterial ‘factory’ for recombinant protein production and a large variety of expression vectors have been created. The vectors may be inducible or inducer-free (constitutive or autoinducible) [[7], [8], [9]]. Inducer-free expression vectors have recently been gaining increased popularity because of the high costs [10] of many inducer compounds and problems with their toxicity [11].

Traditional expression systems utilized high copy number plasmids introduced into *B. subtilis* to create recombinant strains for heterologous protein expression [3]. However, plasmid-less engineered *B. subtilis* strains are preferred in industrial applications due to their stability and lower ecological risk [12]. Formerly, plasmid-free recombinant *B. subtilis* strains were constructed by homologous recombination between the target sequence in the chromosome and the homologous flanking sequences sandwiching the fragment of interest mediating ectopic insertion of the desired genes into the bacterial chromosome [3]. Recently, several new vectors have been developed resulting in more efficient integration. Some of them allow production of recombinant proteins by incorporating many copies of the recombinant gene at different sites in the bacterial genome [5,13]. For example, an integrated vector engineered with the strong inducer-free promoter NBP3510 exhibited β-galactosidase (BgaB) and GFP (green fluorescent protein) production of 43 % and 30 % of the total cellular proteins, respectively [14].

The unsolved problem in the generation of useful vectors allowing robust *B. subtilis* expression systems is their leaky expression in *E. coli.* Most of the expression vectors for *B. subtilis* are shuttle-vectors because the cloning steps must be carried out in *E. coli* [3], which can sometimes result in unexpectedly high protein expression levels [15,16], and, in the worst case, kill the *E. coli* cells. Previous publications did not mention whether strong promoters for *B. subtilis* also cause high background expression in *E. coli.* Here, we constructed inducer-free expression vectors allowing the integration of the recombinant gene into the *B. subtilis* genome. These vectors carried strong promoters and their expression level in *B. subtilis* was comparable to that from high copy number replicative plasmids while expression in *E. coli* remained relatively low. In addition, double-copy insertion strains were generated by the integration of the recombinant gene into two different neutral loci. The best resulting strains had an expression level of over 50 % of the total intracellular proteins.

## 2 Materials and methods

### 2.1 Bacterial strains, plasmids and growth conditions

Plasmids, oligonucleotides, and strains used in this study are shown in Table 1. The *E. coli* strain OmniMAX (Invitrogen) was used as the recipient in all cloning experiments and to determine the background expression levels. All recombinant *B. subtilis* strains used to analyze the expression of the *bgaB* gene were derived from *B. subtilis* 1012. Cultures were initiated from single-colony inocula grown on LB agar plates. Cells were routinely grown in Luria broth (LB) at 37 °C with shaking at 200 rpm. Where necessary, the antibiotics ampicillin at 100 μg/mL for *E. coli* and chloramphenicol at 10 μg/mL for *B. subtilis* were added to recombinant strains harboring replicative plasmids.

**Table 1:** Bacterial strains, plasmids and oligonucleotides used in this study.

Strain or plasmid	Genotype	Source/reference
*E. coli* OmniMAX	*F´ [proAB^+^ lacI^q^ lacZΔM15 Tn10(Tet^R^) Δ(ccdAB)] mcrA Δ(mrr-hsdRMS-mcrBC) Φ80(lacZ)ΔM15 Δ(lacZYA-argF) U169 endA1 recA1 supE44 thi-1 gyrA96 relA1 tonA panD*	Invitrogen
*Bacillus subtilis* 1012	*leuA8 metB5 trpC2 hsdRM1*	[17] and could be obtained at MoBiTec
*B. subtilis* HT2170	Recombinant *B. subtilis* 1012 with the expression cassette of pHT2170 integrated at the *amyE* locus	This study
*B. subtilis* HT2176	Recombinant *B. subtilis* 1012 with the expression cassette of pHT2176 integrated at the *amyE* locus	This study
*B. subtilis* HT2177	Recombinant *B. subtilis* 1012 with the expression cassette of pHT2177 integrated at the *amyE* locus	This study
*Bs/*pHCMC05-*bgaB*	Recombinant *B. subtilis* 1012 carrying pHCMC05-*bgaB*	This study
*Bs*/pHT01-*bgaB*	Recombinant *B. subtilis* 1012 carrying pHT01-*bgaB*	This study
*Bs*/pHT2071	Recombinant *B. subtilis* 1012 carrying pHT2071	This study
*E. coli/* pHT01-*bgaB*	Recombinant *E. coli* OmniMAX carrying pHT01-*bgaB*	This study
*E. coli/*pHT1379	Recombinant *E. coli* OmniMAX carrying pHT1379	This study
*E. coli/*pHT2071	Recombinant *E. coli* OmniMAX carrying pHT2071	This study
*E. coli/*pHT2170	Recombinant *E. coli* OmniMAX carrying pHT2170	This study
pHT01-*bgaB*	P*grac*01*-bgaB, inducible, replicative*	[18]
pHT1305	An empty vector that lacks promoters and reporter genes, carries a neomycin resistance gene and can integrate into the *B. subtilis* chromosome at the *lacA* locus	Lab stock and obtained from Ms. Hanh
pHT1326	An empty vector that lacks promoters and reporter genes, carries neomycin resistance gene flanked by *lox*66 and *lox*71 that can integrate into the *B. subtilis* chromosome at the *lacA* locus	Lab stock and obtained from Ms. Hanh
pHT1379	P*grac*01- multiple cloning site (MCS)*, ΔlacI,* allows integration at the *amyE* locus, served as a negative control	Lab stock and obtained from Ms. Hanh
pHT2071	P*grac*01*-bgaB, ΔlacI,* replicative	[19]
pHT2115	P*grac*01*-bgaB,* intact *lacI*, allows integration at the *amyE* locus	This study
pHT2118	P*grac*100*-bgaB,* intact *lacI,* allows integration at the *amyE* locus	This study
pHT2119	P*grac*212*-bgaB,* intact *lacI*, allows integration at the *amyE* locus	This study
pHT2170	P*grac*01*-bgaB, ΔlacI,* allows integration at the *amyE* locus	This study
pHT2171	P*grac*01-MCS*, ΔlacI,* allows integration at the *lacA* locus, served as a negative control	This study
pHT2172	P*grac*01*-bgaB, ΔlacI,* allows integration at the *lacA* locus	This study
pHT2176	P*grac*100*-bgaB, ΔlacI,* allows integration at the *amyE* locus	This study
pHT2177	P*grac*212*-bgaB, ΔlacI,* allows integration at the *amyE* locus	This study
pHT2184	P*grac*100-MCS, *ΔlacI,* allows integration at the *lacA* locus	This study
pHT2185	P*grac*100*-bgaB, ΔlacI,* allows integration at the *lacA* locus	This study
pHT2188	P*grac*212-MCS*, ΔlacI,* allows integration at the *lacA* locus	This study
pHT2189	P*grac*212*-bgaB, ΔlacI,* allows integration at the *lacA* locus	This study

**Oligonucleotides**	**Sequence** 5’**→** 3’	**Used for**
ON469	GGCGTTCTGTTTCTGCTTCG	To confirm the insertation of the expression cassette at 5’ *amyE*
ON470	AACCCGCTCCGATTAAAGCTAC	To confirm the insertation of the expression cassette at 3’ *amyE*
ON745	CCATGTCTAGAGTCGACGTCAATGTGTTATCCTCAATTTGTTACGG	To confirm the insertation of *bgaB* in *B. subtilis* genome
ON881	TCATGAGCGGATACATATTTGAATGTATTTAG	Colony PCR pHT2185, pHT2189
ON902	GCGCTGATTCATAAATATCTGGTTGTTTC	Colony PCR pHT2170, pHT2176, pHT2177
ON904	GGCCATGACGTCTTCAGGTGTTTCTAAGGAAGGAACG	For sequencing pHT2171
ON941	AAAGGAGGAAGGATCCATGAATGTGTTATC	To amplify *bgaB* gene to construct pHT2185, pHT2189
ON954	GCCTTACAAAATCGACAGCAATATTACG	To confirm the insertation of *bgaB* in *B. subtilis* genome
ON972	TGACGTTATTTCTATATGTATCAAGATAAG	For sequencing pHT2172, pHT2185, pHT2189
ON979	GGCCATGGTCTCGAATTAGATAAAAAATTTAGAAGCCAATGAAATC	To confirm the insertation of the expression cassette at 5’ *amyE*
ON1207	GGGAGATTCTTTATTATAAGAATTG	For sequencing pHT2172, pHT2189
ON1249	CGTTTCCACCGGAATTAGCTTG	Colony PCR pHT2171
ON1354	AACACCAATAGCCTTAACATCATCC	Colony PCR pHT2171
ON1441	CTATACGACATTTGCGGCCGGAG	To confirm the insertation of the expression cassette at 5’ *lacA*
ON1442	GATCCTCTGCCCGAAGCTCTGAC	To confirm the insertation of the expression cassette at 3’ *lacA*
ON1479	GTCTGGTCAACTTTCCGACTCTG	To confirm the insertation of the expression cassette at 5’ *lacA*
ON1896	CGGTTCGATCTTGCTCCAACTG	Colony PCR pHT2170, pHT2172, pHT2176, pHT2177 To confirm the insertation of the expression cassette at 3’ *lacA*
ON1975	CAATTGCGTTGCGCTCACTGCCAGCGCT	To create *lacO3* in pHT2170, pHT2176, pHT2177
ON1976	AGCGCTGGCAGTGAGCGCAACGCAATTGAGCT	To create *lacO3* in pHT2170, pHT2176, pHT2177 To confirm the insertation of the expression cassette at 3’ *amyE*
ON2134	GCCCCGGGGACGTCCTAAACCTTCCCGGCTTCATC	To amplify the *bgaB* gene to construct pHT2185, pHT2189
ON2194	GGCCATGAATTCACTAGTGATCTCCATGGACGCGTGACG	To amplify P*grac*01 to construct pHT2171
ON2195	GGCCATAGATCTATCGATTCGAGCTCAATTGCGTTGCGCTCAC	To amplify P*grac*01 to construct pHT2171

*ΔlacI*, deletion of partial or full *lacI* gene; P*grac*01 (another name is P*grac*) [20]; P*grac*100 [21] and P*grac*212 [18] are the names of different promoters. Fig. 4**D** shows the alignment of the promoter sequences.

### 2.2 Construction of integrative vectors

#### 2.2.1 Expression vectors integrating into the lacA locus

We first created the three basic inducer-free integrative expression vectors, pHT2171, pHT2184 and pHT2188, and then, the *bgaB* gene was introduced into these vectors to obtain pHT2172, pHT2185 and pHT2189. pHT2171 was constructed by inserting the cassette containing the *lacO3* operator and the P*grac*01 promoter (amplified from pHT2134 as the template, a derivative of pHT2071 [19] carrying a new MCS, with ON2195/ON2194) into the pHT1305 empty vector which contains sequences to allow integration at the *lacA* locus on the *B. subtilis* genome without any promoter. pHT2184 was constructed by insertion of the cassette harboring *lacO3* and the P*grac*100 promoter into the pHT1326 backbone. This backbone has no promoter but contains the homologous sequences allowing integration into the *lacA* locus on the *B. subtilis* genome. pHT2184 was first cleaved with *Sac*I and *Bam*HI to remove P*grac*100. Then, it was ligated to P*grac*212 amplified using the pHT2080 template to create pHT2188.

#### 2.2.2 Inducer-free integrative vectors containing Pgrac01-bgaB, Pgrac100-bgaB, and Pgrac212-bgaB

To construct pHT2172, pHT2185, and pHT2189, we amplified the *bgaB* gene using the primer pairs ON2134 and ON941 with pNDH33-*bgaB* as a template. The *Bam*HI/*Aat*II-treated PCR product was introduced into pHT2171, pHT2184, and pHT2188 at the *Bam*HI and *Aat*II sites, respectively.

#### 2.2.3 Expression vectors able to integrate into the amyE locus

To construct plasmids pHT2170, pHT2176, and pHT2177, we removed *lacI* together with the *lacO3* sequence from plasmids pHT2115, pHT2118, and pHT2119, respectively, and inserted the *lacO3* sequence between the *Sna*BI and *Sac*I restriction sites by using the two complementary oligonucleotides ON1975 and ON1976.

### 2.3 Generation of *E. coli* and *B. subtilis* recombinant strains

Expression vectors were confirmed by DNA sequencing. The correct vectors were transformed into *E. coli* OmniMAX as described in [22] and into *B. subtilis* 1012 as described elsewhere [23]. Recombinant *B. subtilis* strains generated by double crossover events were screened by PCR using specific oligonucleotide pairs. Fig. 2**A** and **D** shows specific primers with the length of the PCR products for the strains carrying P*grac*01 that integrate into *amyE* and *lacA* loci. The same PCR approach has been used to confirm the integration of the expression cassettes containing P*grac*100 and P*grac*212.

### 2.4 Measurement of BgaB expression levels in *E. coli* and *B. subtilis*

Recombinant strains were streaked on LB agar plates with the appropriate antibiotic: ampicillin at 100 μg/mL for *E. coli* and chloramphenicol at 10 μg/mL for *B. subtilis* harboring replicative plasmids, and no antibiotic for *B. subtilis* strains carrying integrated sequences. A single colony was inoculated into a culture tube containing 5 mL LB medium and antibiotic and shaken overnight at 200 rpm at 37 °C. Cultures of each strain were replicated using three separate colonies. The OD_600_ of the pre-culture was measured and an appropriate volume of pre-culture of each clone was transferred to 30 mL LB medium containing the appropriate antibiotic in 100 mL shake flasks to give an OD_600_ of 0.1 and incubated with shaking at 37 °C. When the OD_600_ of the culture reached 0.8–1, the cells were divided into two sub-cultures and one of them was induced by the addition of IPTG to a final concentration of 1 mM. Cells were collected by centrifugation at 0 h just before induction and at 2, 4, 6, 8, 10, 12 h after induction. The OD_600_ of all sub-cultures was monitored and a volume equivalent to an OD_600_ of 2.4 was pipetted into 1.5 mL Eppendorf tubes, centrifuged, and the supernatants removed. Samples were prepared for activity measurements and for SDS-PAGE analysis. BgaB activity was measured as described in [19].

For SDS-PAGE analyses, cell pellets were lysed by the addition of 100 μL lysis buffer (25 mM SDS, 250mM sucrose) with an addition of 2.5 μL lysozyme at 50 mg/mL. The mixtures were vortexed thoroughly and incubated at 37 °C for 5 min. After that, 25 μL of 5X sample buffer (10 mL Tris −HCl pH 6.8, 1.54 g dithiothreitol, 0.4 g SDS, 4 mL glycerol, bromophenol blue, and dH_2_O up to 20 mL) was added. The samples were mixed well and heated at 95 °C for 5 min followed by centrifugation at 15871 rcf for 5 min. Aliquots of 8 μL of each sample were applied to each well on SDS-PAGE gels [24].

## 3 Results

### 3.1 Background expression levels of P*grac*01-*bgaB* constructs in *E. coli*

Expression vectors for *B. subtilis* are shuttle-vectors, and the cloning steps are carried out in *E. coli.* However, a vector that could drive a high level of expression in *B. subtilis* often results in a high level of expression in *E. coli* even in the absence of inducers. Previously, we reported a new strategy to construct inducer-free plasmids based on IPTG-inducible promoters. By deleting part of the *lacI* gene in IPTG-inducible pHT vectors, the resulting plasmids could express the target proteins in the absence of IPTG in *B. subtilis* while still repressing the background expression in *E. coli* through the chromosomal *lacI* gene [19]. To generate expression vectors for *B. subtilis* which express the recombinant protein at high levels in *B. subtilis* while performing very low expression in *E. coli*, we used this strategy to develop inducer-free integrative expression vectors based on P*grac* promoters as reported earlier [19]. The promoters are flanked by two *lac* operators, *lacO1* at the downstream and *lacO3* at upstream of the promoters, which promote the repression of leaky expression by DNA-loop formation in the presence of the LacI repressor [25]. Besides, to increase the stability of the vectors as well as to lower the background expression in *E. coli,* we introduced the *rop* gene into the vectors. The Rop protein decreases the copy number of ColE1-like plasmids by stabilizing the RNAI-RNAII duplex [26].

An integrative expression vector harboring the P*grac*01 promoter and the *bgaB* reporter gene was constructed, and the leaky expression level was compared with that of some other replicative plasmids.

**Fig. 1:**
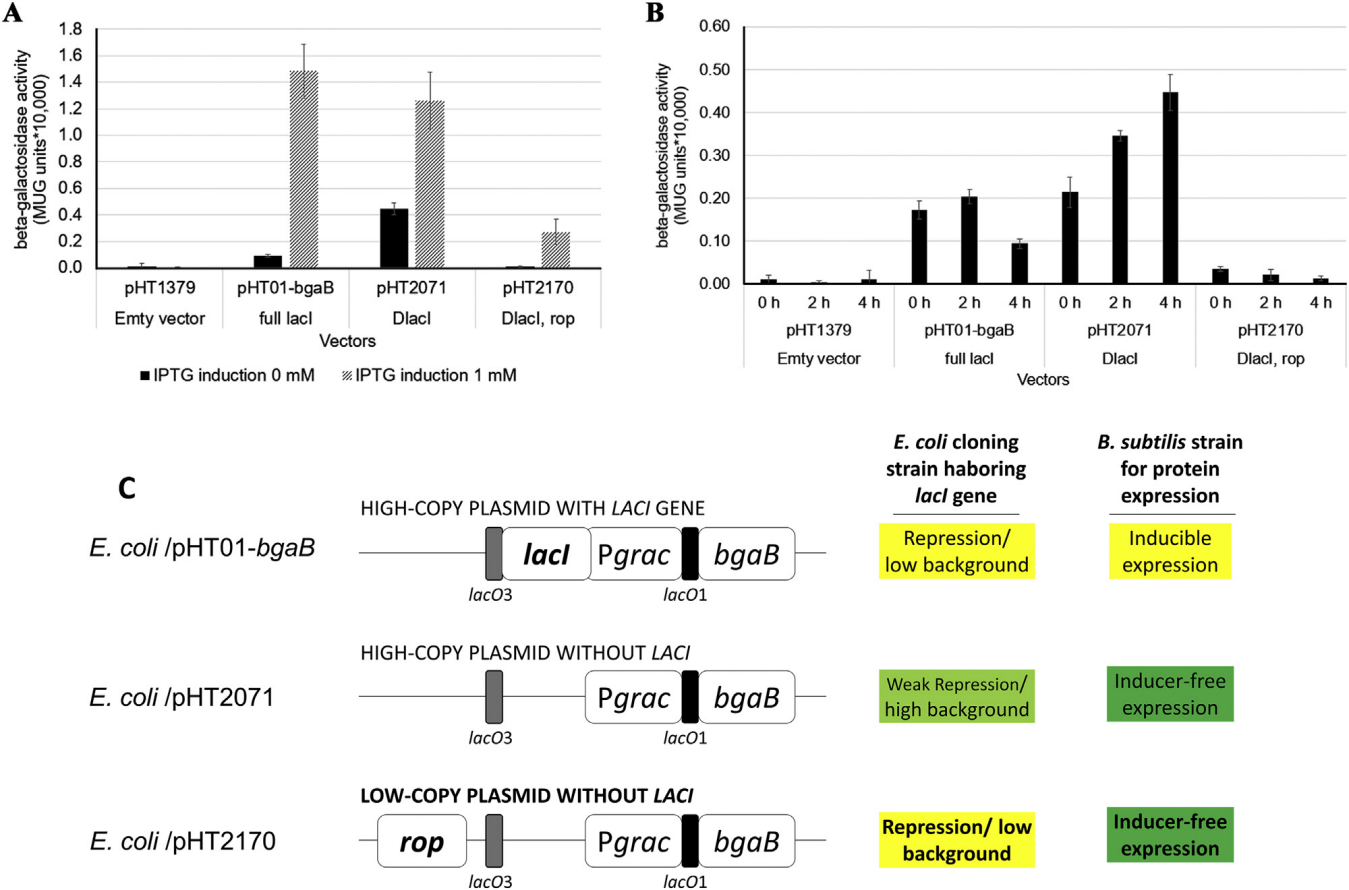
**Background expression of *bgaB* constructs in *E. coli* OmniMAX and the concept of the repression of the P*grac* by *lacI* and plasmid-copy number.** (A) IPTG induction. (B) Leaky expression in the absence of IPTG. pHT1379 (P*grac*01 without the *bgaB* gene served as a negative control), pHT01-*bgaB* (P*grac01,* replicative, IPTG-inducible), pHT2071 (P*grac01,* replicative, inducer-free), pHT2170 (P*grac*01, integrative, inducer-free). Recombinant strains were cultured separately in LB medium with ampicillin at100 μg/mL until mid-log phase, after which the cultures were divided into subcultures and induced with 1 mM IPTG. Controls received no IPTG. Samples were harvested at 0 h just before induction and 2 h and 4 h after induction. (C) The conceptual figure of the repression of the P*grac* promoter by plasmid and chromosomal *lacI* and control of plasmid copy via *rop* gene in *E. coli* and types of expression in *B. subtilis* expression host.

While pHT01-*bgaB* (P*grac*01*-bgaB,* containing *lacI*) could repress leaky expression in *E. coli* OmniMAX about 16-fold, pHT2071 (P*grac*01*-bgaB,* Δ*lacI*) could repress it only about 3-fold, and pHT2170 (P*grac*01*-bgaB,* Δ*lacI, rop*) could repress it about 24-fold (Fig. 1A). These results demonstrate that the combination of two *lac* operators together with the *rop* gene in the pHT2170 plasmid created optimal repression of background expression in *E. coli*. The leaky expression of the *bgaB* gene from the inducer-free integrative expression vectors was the lowest among these vectors analyzed (Fig. 1B). Moreover, the leaky expression from pHT2170 was approximately similar to pHT1379 which served as the negative control which did not express *bgaB*. These results demonstrated that pHT2170 could repress the leaky expression in *E. coli* thereby allowing the cloning steps. The conceptual figure (Fig. 1C) shows the repression of the P*grac* promoter by plasmid and chromosomal *lacI* and control of plasmid copy via *rop* gene in *E. coli* cloning strain.

### 3.2 Inducer-free expression of P*grac*01-*bgaB* integrated into the *B. subtilis* genome

**Fig. 2:**
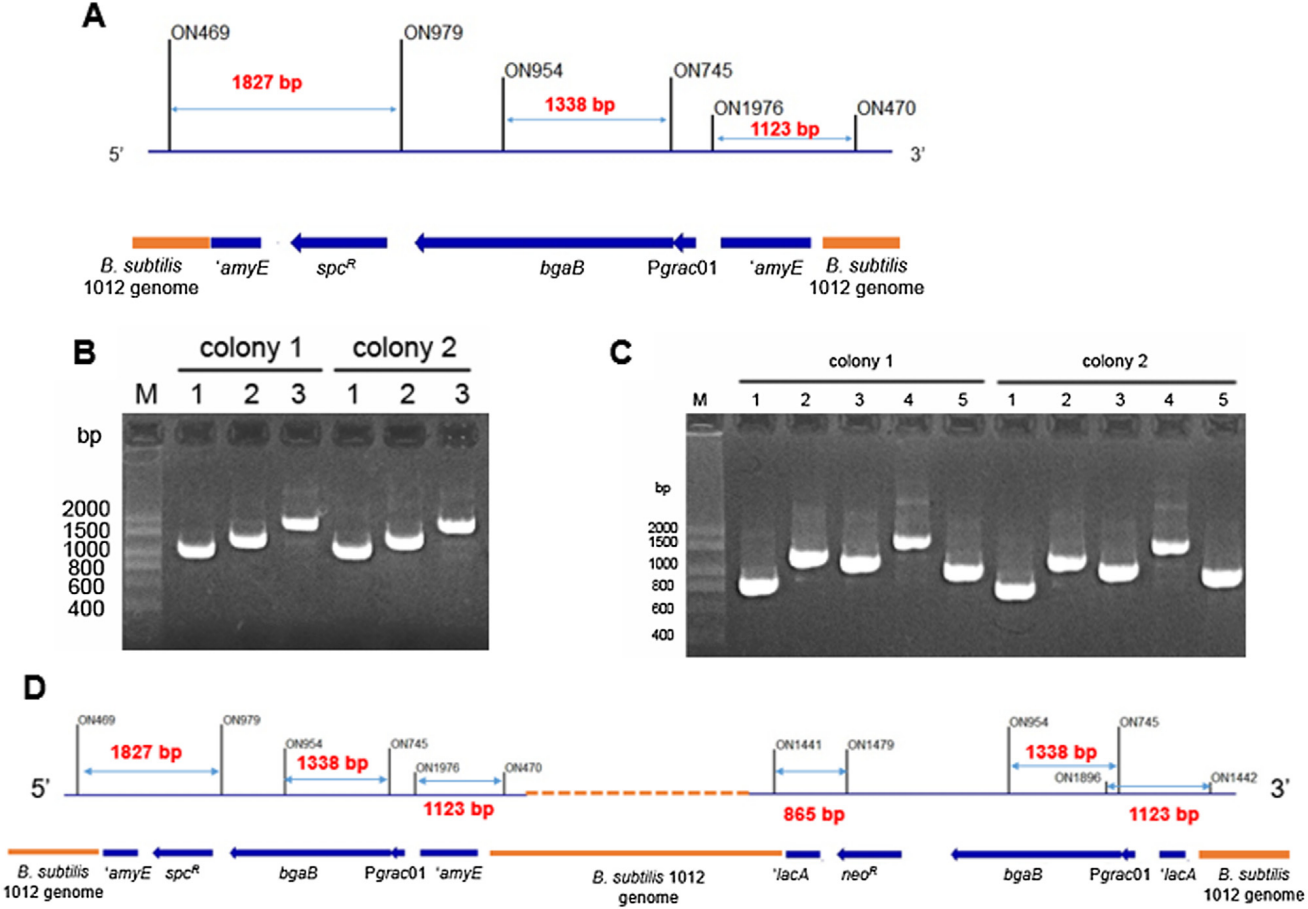
**Confirmation of integration by PCR.** (A) Schematic diagram shows the sites of oligonucleotides used for checking the double crossover at both *amyE* in *B. subtilis* recombinant strains (B) Electrophoresis of PCR products using *B. subtilis* HT2170 as template on 2% agarose gel, 1: PCR products by ON1976/ON470, 2: PCR products by ON954/ON745, 3: PCR products by ON469/ON979 (C) Electrophoresis of PCR products of *B. subtilis* strain with integration at both *amyE* and *lacA* loci on 2% agarose gel, 1: PCR products by ON1441/ON1479, 2: PCR products by ON954/ON745, 3: PCR products by ON1896/ON1442, 4: PCR products by ON469/ON979, 5: PCR products by ON1976/ON470 (D) Schematic diagram shows the sites of oligonucleotides used for PCR to check the double crossover at both *amyE* and *lacA* loci in *B. subtilis* recombinant strains. The length of the PCR products are indicated in the Figure.

**Fig. 3:**
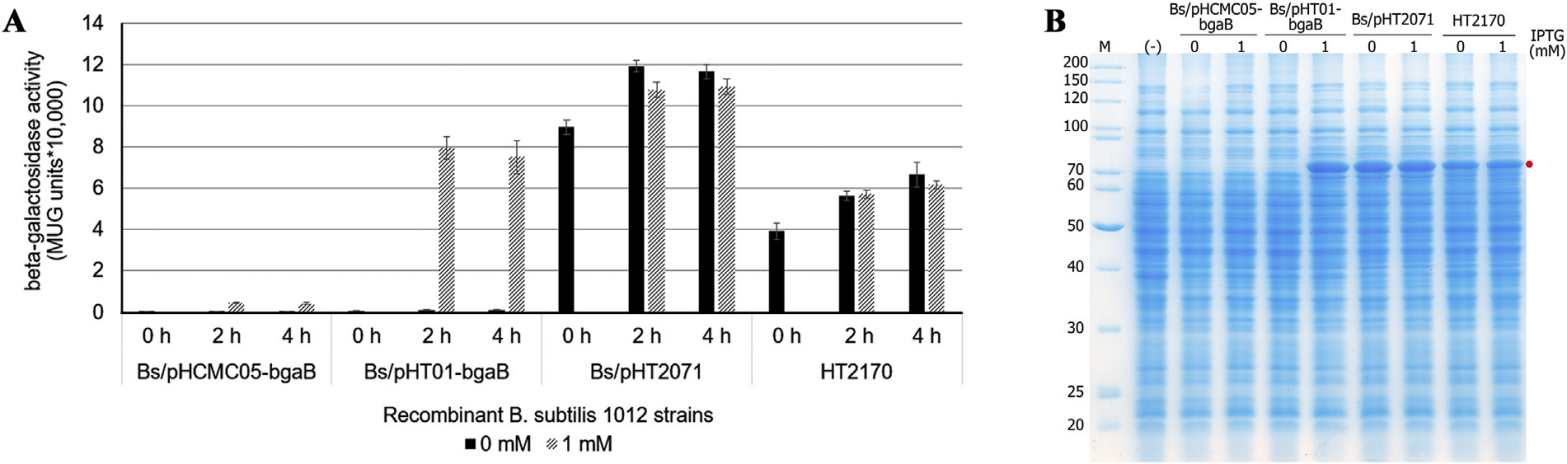
**Inducer-free expression of P*grac*01-*bgaB* integrated into the *B. subtilis* 1012 genome in comparison with replicative plasmids.** (A) BgaB activities (B) SDS-PAGE. pHCMC05*-bgaB* (P*spac,* replicative, IPTG-inducible), pHT01-*bgaB* (P*grac01,* replicative, IPTG-inducible), pHT2071 (P*grac*01, replicative, inducer-free), pHT2170 (P*grac*01, integrative, inducer-free). Recombinant strains were cultured in LB medium without antibiotic (pHT2170) or with chloramphenicol (Cm) at 10 μg/mL (pHCMC05-*bgaB*, pHT01-*bgaB*, HT2071) until mid-log phase, after which the cultures were divided into sub-cultures and induced with 1 mM IPTG. Controls received no IPTG. Samples were harvested at 0 h just before induction and 2 h and 4 h after induction. Proteins were extracted from aliquots of 4 h cultures and used for SDS-PAGE.

The expression cassette of the vector pHT2170 consisted of two homologous sequences of the *amyE* gene from *B. subtilis* genome, the *spc^R^* gene for selection, the P*grac01* promoter and the *bgaB* reporter gene. This vector does not include an origin of replication for *B. subtilis*; therefore, when it is transformed into *B. subtilis* cells in the presence of spectinomycin, the expression cassette is integrated into the genome by either a single or a double crossover event. The latter was confirmed by PCR (Fig. 2A, B) and the resulting strain was named *B. subtilis* HT2170. The expression cassette was stably maintained in the genome of this strain even in the absence of spectinomycin. The *bgaB* expression level in *B. subtilis* HT2170 in the absence of IPTG and antibiotics was tested and compared with those of some other replicative plasmids carrying the same or a different promoter. HT2170 synthesized the BgaB protein in a manner different from strains carrying pHT01-*bgaB* or pHCMC05-*bgaB*, but in the same way as with pHT2071. While pHT01-*bgaB* and pHCMC05-*bgaB* expressed *bgaB* only in the presence of IPTG, pHT2170 synthesized BgaB similarly in the absence or presence of IPTG (Fig. 3A). SDS-PAGE analyses (Fig. 3B) confirmed this result. These results indicate that pHT2170 can efficiently express *bgaB* in an inducer-free manner. The best performance of pHT2170 was 6.66 × 10^4^ methylumbelliferyl β-d-galactopyranoside (MUG) units while that of pHCMC05-*bgaB* with the P*spac* promoter reached only 0.47 × 10^4^ MUG units. The BgaB activity of pHT2170 was about 14-fold higher than that obtained with the multi-copy replicative P*spac* plasmid. The SDS-PAGE results (Fig. 3B) also confirmed that the expression level of the inducer-free integrative vector pHT2170 was much higher than that of pHCMC05-*bgaB*. However, the BgaB activity of pHT2170 was lower than that obtained with replicative plasmids carrying the same promoter. The BgaB activity of pHT2170 was about 80 % of pHT01-*bgaB* after induction with 1 mM IPTG and about 50 % of that obtained with pHT2071 (Fig. 3A). In addition, SDS-PAGE analysis revealed that BgaB expressed by pHT2170 accounted for only 8% of the total protein while pHT2071 expressed 14 %, and pHT01-*bgaB* about 10 % (Fig. 3B).

The expression of *B. subtilis* HT2170 was 1.25 or 1.75 times less than plasmid expression carrying the same promoter, most probably because of the lower copy number. Therefore, to generate integrative vectors whose expression level was comparable with that of multi-copy plasmids, we next constructed integrative vectors with stronger promoters.

### 3.3 High expression levels of the *bgaB* reporter gene using stronger P*grac* promoters

**Fig. 4:**
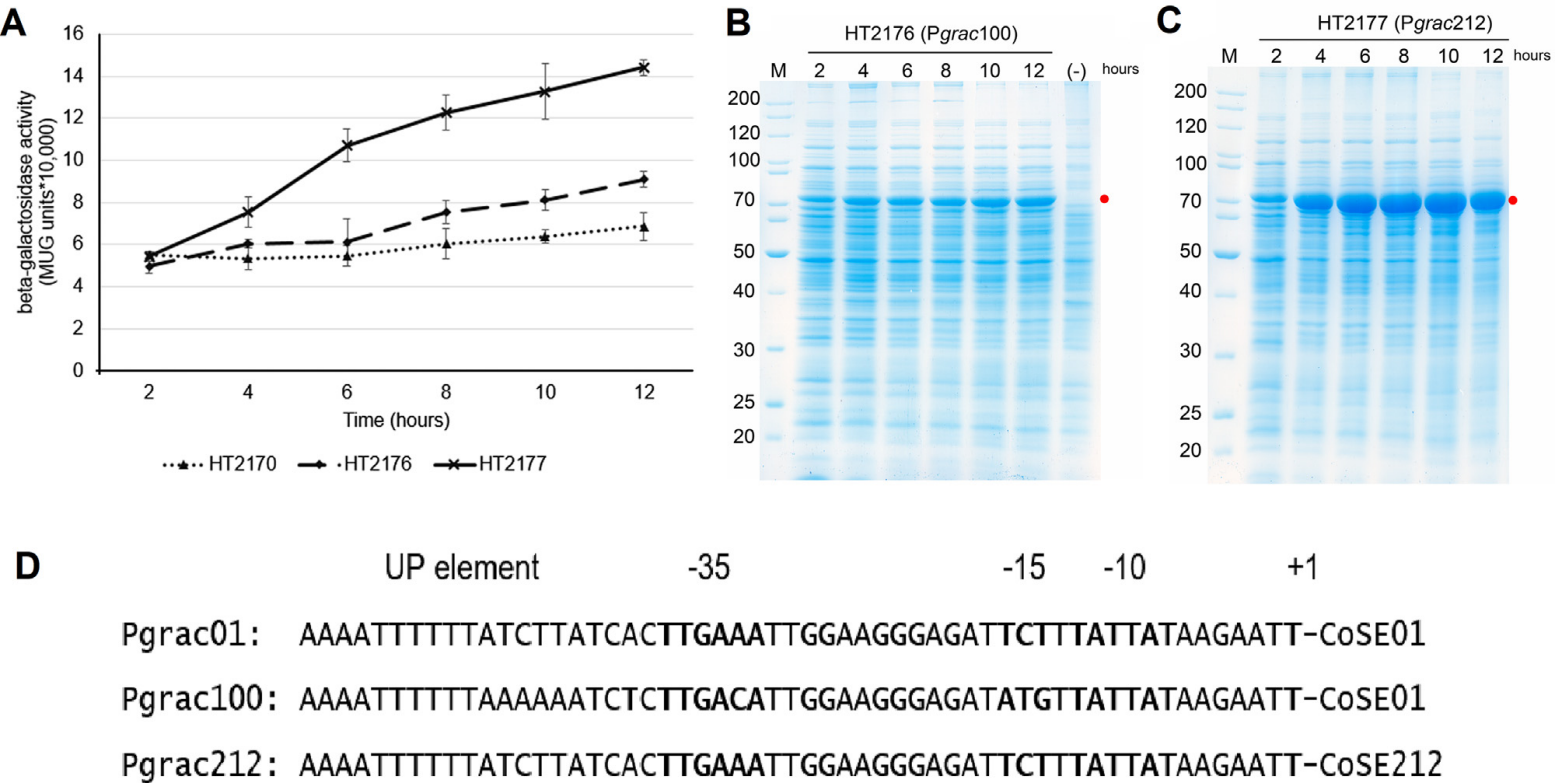
**High expression of BgaB from a single copy of an integrative expression cassette carrying strong P*grac* promoters in *B. subtilis* 1012.** (A) BgaB activity of vectors integrated at the *amyE* locus (B) SDS-PAGE of pHT2176 (P*grac*100) (C) SDS-PAGE of pHT2177 (P*grac*212) (D) Sequences of P*grac*01, P*grac*100, P*grac*212 promoters. Recombinant strains were cultured in LB medium without antibiotics until mid-log phase. Samples were harvested at 0 h when the OD_600_ reached 0.8-1 and 2 h and 4 h later. Samples obtained 4 h after induction were used for SDS-PAGE analyses.

Next, we aimed to construct inducer-free integrative vectors with high-level expression by using the strongest promoters from the P*grac* library. On example is the engineered P*grac*100 promoter whose UP element, the -35 and -15 sequences have been mutated allowing intracellular accumulation of BgaB up to 30 % [27]. P*grac*212 is structurally similar to P*grac*01 containing modifications at the controllable stabilizing element (CoSE; the region from +1 to the RBS) [15] resulting in BgaB levels within the same range as compared to P*grac*100 [21]. Therefore, P*grac*100 and P*grac*212 were chosen. The highest BgaB activities were 6.9 × 10^4^ MUG units for the vector carrying the P*grac*01 promoter, 9.1 × 10^4^ MUG units for those with P*grac*100, and 14.4 × 10^4^ MUG units for those with P*grac*212 (Fig. 4A). Using the stronger promoters, *bgaB* expression increased 1.3 up to 2.1-fold. Analysis of Fig. 4B and 4C by Alpha Ease 4.0 showed that BgaB expressed by P*grac*100 vectors accounted for 20.9 % and P*grac*212 vectors accounted for 42 % of the total intracellular proteins after 12 h. BgaB expressed by HT2176 -P*grac*100 accounted for 9% of the total cellular protein at 2 h, 12 % at 4 h and 20.9 % at 12 h (Fig. 4B). Similarly, the amount of BgaB produced by HT2177-P*grac*212 was low at the early stage of the culture, but rose rapidly with time-11 % of the total protein at 2 h, 32.7 % after 4 h, finally reaching 42 % after 12 h, or about 4-fold over time in culture (Fig. 4C).

### 3.4 Increasing expression levels by integrating P*grac*212-*bgaB* into both *amyE* and *lacA* loci

Another way to increase the expression level in *B. subtilis* strains carrying the expression unit in the genome is to increase the copy number of the expression unit. Several methods are available for multi-copy insertions of the target gene. Among them, integration at two and more different chromosomal sites may be the most stable [28], so the expression cassette was inserted into both the *amyE* and the *lacA* loci to increase the magnitude of expression of the target gene. The vector was first transformed into competent *B. subtilis* cells and the integration occurred at the *amyE* locus in the presence of spectinomycin. Next, a second vector with homologous sequences to the *lacA* locus was transformed into the strain already carrying one copy of the expression unit at the *amyE* locus in the presence of neomycin. The recombinant strains possessed two expression cassettes in the genome were confirmed by PCR (Fig. 2C, D), and *bgaB* expression was evaluated by the MUG assay and by SDS-PAGE.

**Fig. 5:**
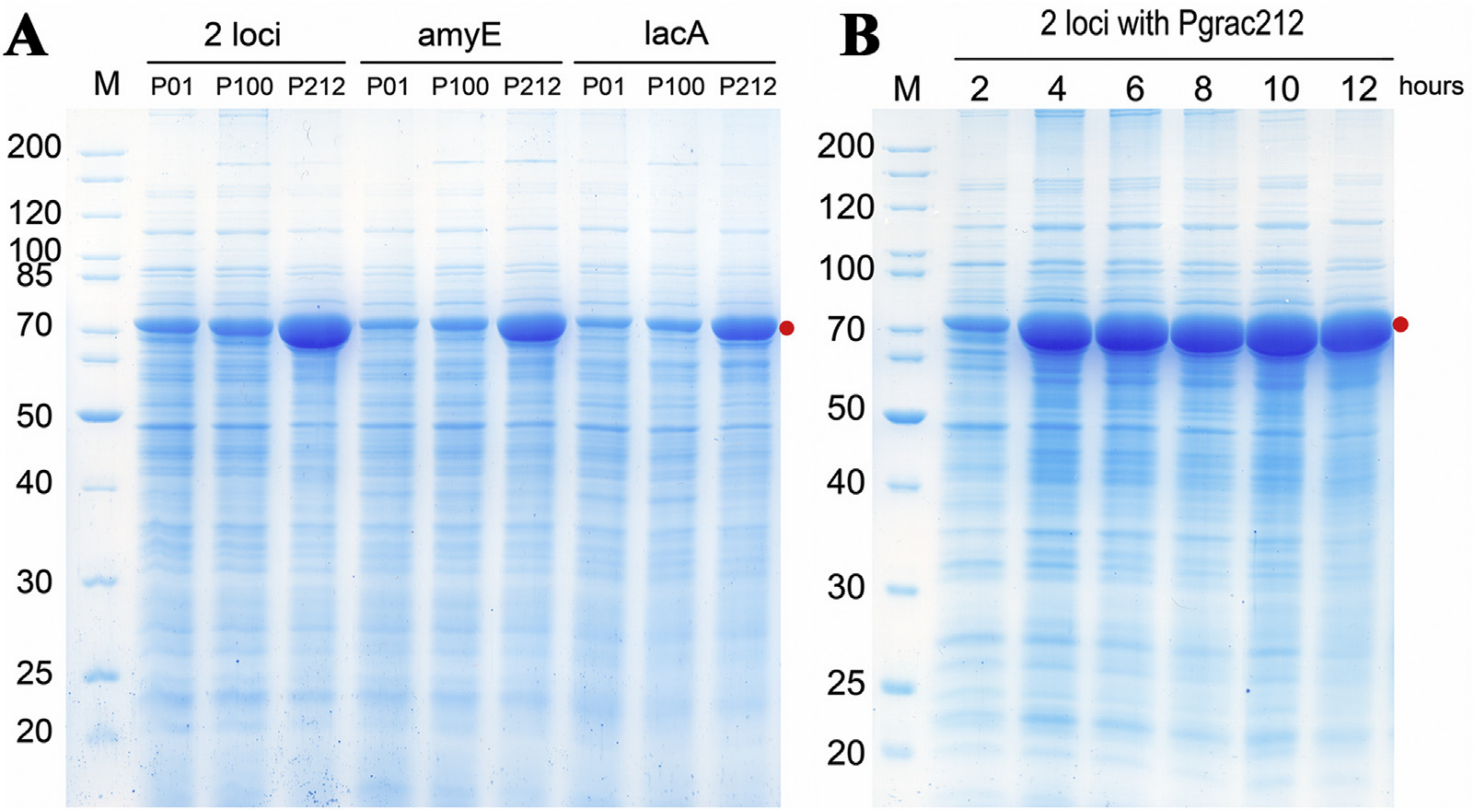
**Increasing expression levels of BgaB by integrating constructs at both *amyE* and *lacA* loci in the *B. subtilis* 1012 genome.** (A) SDS-PAGE with different promoters; P01, P*grac*01, P100, P*grac*100, P212, P*grac*212; (B) SDS-PAGE with P*grac*212-*bgaB* integrated at both *amyE* and *lacA* loci. Recombinant strains were cultured in LB medium without antibiotics until the mid-log phase. Samples were harvested at 0 h (when OD_600_ reached 0.8-1) and 2 h - 12 h later.

Integration of the *bgaB* gene into both the *amyE* and the *lacA* loci doubled the copy number of the gene and the synthesis of BgaB from both loci was much higher than the expression from either the *amyE* or the *lacA* locus under control of the same promoter. Strains with expression cassettes containing P*grac*01, P*grac*100, or P*grac*212 integrated at the two loci expressed BgaB at levels of 23.4 %, 24 %, and 53.4 %, of total proteins, respectively. The expression of BgaB was increased from 1.1 to 1.3-fold (Fig. 5A). The strain with P*grac*212 expression cassettes inserted into both loci synthesized BgaB at 11 % after 2 h of induction, 34.7 % after 4 h and continued increasing to 53.4 % at 12 h (Fig. 5B).

## 4 Discussion

Due to its many favorable characteristics, *B. subtilis* serves as an excellent cell factory for the production of heterologous proteins. However, competent *B. subtilis* cells for cloning experiments are present only in low numbers resulting in poor transformation efficiency. The competence problem has been overcome by performing the cloning steps in *E. coli* using *B. subtilis – E. coli* shuttle vectors [3], but leaky protein expression in *E. coli* can hamper the cloning efficiency. The strong promoters that allow a high level of expression in *B. subtilis* also drive leaky expression to high levels in *E. coli.* Many regulatable vectors for strong protein synthesis in *B. subtilis* result from the incorporation of a promoter with the *lac* operator [[29], [30], [31], [32]]. This type of hybrid promoter is used in IPTG-inducible vectors to prevent unwanted expression in *E. coli*. However, the difference between the presence of the *lacI* gene in *B. subtilis* and in the *E. coli* genome allows *lacO*-hybrid promoters to be used to create vectors that are inducer-free in *B. subtilis* but remain controllable in *E. coli.* In a previous study, we reported a new strategy for generating inducer-free replicative plasmids [19] to achieve high-level expression in *B. subtilis* while suppressing leakiness in *E. coli*; but the level of background expression in *E. coli* still remained high. These vectors were based on ColE1-like plasmids and the *rop* gene could be used to reduce the expression of leaky promoters in *E. coli* by lowering the plasmid copy number. The gene encoding the Rop protein is removed in most vectors to increase their copy number in *E. coli* [33], but we chose to introduce it into our vectors because they were constructed for recombinant protein synthesis in *B. subtilis* where low plasmid copy number in *E. coli* would not be a problem. On the contrary, it helps to reduce background expression in this host. In this study, the combination of the *rop* gene and the two *lac* operators in our vectors led to 24-fold repression in recombinant *E. coli* carrying pHT2170 with the P*grac*01 promoter compared to only 3-fold with the previously reported strategy, significantly decreasing the background expression. After 4 h, the leaky BgaB was only around 230 MUG units, 19-fold lower than with pHT2071 (P*grac*01, replicative, inducer-free, high copy number) and 4-fold lower than pHT01-*bgaB* (P*grac*01, replicative, IPTG-inducible, high copy number). The conceptual figure (Fig. 1C**)** shows the background in *E. coli* and the inducible or inducer-free expression in *B. subtilis* in the host.

Replicative plasmids can have problems with stability and safety however, so bioengineers are turning more to vectors integrated into the host’s genome. A single integrated expression cassette may not produce the desired expression level, so efforts are being made to increase the copy number in the genome in order to boost expression. Ten copies of an *mpr^B.amy^* cassette in which the GSP gene was placed between the promoter of the *B. amyloliquefaciens rplU-rpmA* gene and the Rho-independent transcription terminator were ectopically inserted into designated (3 copies) and random (7 copies) sequences into the recipient’s DNA. The resulting bacterial strain produced approximately 0.5 g/L of secreted GSP after cultivation in flasks with starch-containing media, and its performance was comparable to an analogous strain in which the *mpr^B.amy^* cassette was carried on a multi-copy plasmid [12]. In another study, nine copies of a *arg^R.pyc^* cassette containing the *Rummeliibacillus pycnus* arginase gene regulated by the strong promoter P43 were inserted into the recipient’s genome. Tests showed that the highest arginase activity (14.5 U/mL) was obtained from flask cultures, and this segregation-stable strain could efficiently hydrolyze l-arginine with a 97.2 % molar yield, suggesting a potential application for the food industry [28]. A strong promoter was engineered that allowed synthesis of BgaB and sfGFP to levels of 43 % and 30 % of intracellular proteins, respectively. It was also used to allow the secretion of methyl parathion hydrolase (MPH) and chlorothalonil hydrolytic dehalogenase (Chd) to a level of 0.3 g/L (144 U/mL) and 0.27 g/l (4.4 U/mL) using shake-flask culture conditions [14]. In our study, the strong P*grac* promoters were used to generate effective *B. subtilis – E. coli* inducer-free integrative vectors. The best performance of plasmid-less strain with a single genomic copy of the BgaB expression cassette produced target protein to 42 % of total protein. With ectopic insertion into both *amyE* and *lacA* loci, the BgaB yield reached 53.4 % of total protein. A series of different integrative vectors with a variety of expression levels were created to meet different protein expression needs.

Inducer-free expression vectors (constitutive and auto-inducible) avoid the need to add an inducer to the culture medium thereby lowering the production cost. Constitutive promoters are not suitable for the production of toxic proteins, but auto-inducible promoters are ideal for large-scale commercial protein production. Such promoters induce expression of the target gene from the late log phase to the stationary phase with no requirement for an inducer, which facilitates high-yield production of heterologous proteins at low cost [34]. The silencing of the *lacI* gene in designated vectors allowed constitutive expression in *B. subtilis*. The best performance was obtained with vectors expressing the target protein at low levels during early culture stages but switching to high production when host cells reach the late log phase. As demonstrated here, *B. subtilis* carrying the P*grac*212 cassette expressed BgaB protein only up to 11 % during the first two hours after induction, then the yield increased to 30 % over the next two hours when the cells begin to enter stationary phase. These positive results show the potential commercial value of our newly constructed vectors. These efforts also constitute an extension of our previous investigation of *B. subtilis* as a vaccine delivery vector. We showed that the expression of small amounts of LTB in the cytoplasm or anchored on the cell surface by a sortase [35,36] could induce a humoral immune response in mice [37]. The use of a vector carrying P*grac*212 linked to a gene encoding the human rhinovirus 3C protease resulted in the production of recombinant protein up to 16 % in *B. subtilis* after IPTG induction [38]. Recent reports using different expression systems showed the expression of various recombinant proteins in *B. subtilis* such as α-amylase, PhoA, single-chain variable antibody fragment, RNase barnase, trehalose synthase, human FGF21 [[39], [40], [41]]. Therefore, our inducer-free expression system could be a cost-effective solution for synthesizing recombinant proteins or vaccines for animals.

## Funding sources and acknowledgements

This work was supported by the Vietnam National Foundation for Science and Technology Development (NAFOSTED) under grant number 106-NN.02-2015.24. The equipment was provided by TWAS under research grant 14–201 RG/BIO/AS_G, TWAS. W. Schumann would like to thank the SES for financial support to travel to Vietnam. The authors would like to thank Ms Hanh for kindly providing plasmid pHT1305, pHT1326, and pHT1379 for this study.

## CRediT authorship contribution statement

**Dinh Thi Minh Tran:** Investigation, Writing - original draft. **Trang Thi Phuong Phan:** Investigation, Resources, Project administration. **Thanh Thi Ngoc Doan:** Validation, Resources. **Thuoc Linh Tran:** Supervision. **Wolfgang Schumann:** Writing - review & editing. **Hoang Duc Nguyen:** Conceptualization, Supervision, Methodology.

## Declaration of Competing Interest

The authors report no declarations of interest.
